# Supplementary material for: Virulence Mechanisms of Common Uropathogens and Their Intracellular Localisation within Urothelial Cells
Source: Pathogens. 2022 Aug 17;11(8):926. doi: 10.3390/pathogens11080926 (PMC9415470; doi:10.3390/pathogens11080926)
Supplement: Supplementary file 1 [file pathogens-11-00926-s001.zip › pathogens-1859069-supplementary.pdf]

Supplementary

Table S1. A detailed summary of all bacterial isolates and the patients they were obtained from.

Bacterial Species	Isolate Name	Site of Specimen	Susceptible	Resistant	Patient Notes
Escherichia coli	EC1	MSU	AMI, AMC, CEP, COT, CTX, CLX, GEN, IMI, NFT, NOR, TMP	AMX, SFZ	90 y/o; TKR; uterine prolapse.
Escherichia coli	EC2	MSU	AMI, AMC, CEP, COT, CTX, CLX, GEN, IMI, NFT, NOR	TMP, SFZ	78 y/o; febrile; bone marrow biopsy; previous UTI history.
Escherichia coli	EC3	MSU	AMI, CEP, COT, CTX, CLX, IMI, NFT	AMX, AMC, AMP, GEN, NOR, SFZ, TMP	52 y/o; renal transplant recipient with IgA nephropathy.
Enterococcus faecalis	EF1	MSU	AMX, AMP, DOX, NFT, VAN	TTC	94 y/o; had neck of femur fixed with IDC inserted 2 weeks prior to MSU.
Enterococcus faecalis	EF2	IDC	AMX, AMP, DOX, NFT, VAN	TTC	61 y/o; mesh repair for recurrent ventral hernia 5 days prior to IDC specimen; symptomatic UTI.
Enterococcus faecalis	EF3	SPC	AMX, AMP, DOX, NFT, VAN	No resistances recorded	66 y/o; symptomatic UTI; post-op SPC in situ for 8 days; had DO.
**Streptococcus sp., Group B**	GBS1	MSU	AMX, AMP, NFT, PEN, VAN	DOX, TTC	82 y/o; severe arthritis; recurrent UTI.
**Streptococcus sp., Group B**	GBS2	MSU	AMX, AMP, DOX, NFT, VAN	TTC	64 y/o; renal transplant recipient.
**Streptococcus agalactiae (Group B)**	GBS3	MSU	AMX, AMP, DOX, NFT, PEN, TTC, VAN	No resistances recorded	29 y/o; history of UTIs; urinary urge and stress incontinence; painful bladder; symptomatic UTI.

Antibiotics: AMI, Amikacin; AMX, Amoxicillin; AMC, Amoxicillin and Clavulanic acid; AMP, Ampicillin; CEP, Cefepime; COT, Cefotaxime; CTX, Ceftriaxone; CLX, Cephalexin; DOX, Doxycycline; GEN, Gentamycin; IMI, Imipenem; NFT, Nitrofurantoin; NOR, Norfloxacin; SFZ, Sulfafurazole; TTC, Tetracycline; TMP, Trimethoprim; VAN, Vancomycin. DO, detrusor overactivity; IDC, indwelling catheter; MSU, mid-stream urine; post-op, post-operation; SPC, suprapubic catheter; TKR, total knee replacement; UTI, urinary tract infection.
